# Stress response induced by carbon nanoparticles in Paracentrotus lividus

**Published:** 2012

**Authors:** Elisabetta Carata, Bernardetta Anna Tenuzzo, Federica Arnò, Alessandro Buccolieri, Antonio Serra, Daniela Manno, Luciana Dini

**Affiliations:** 1 *Department of Biological and Environmental Sciences and Technologies, University of Salento, Lecce, Italy*; 2 *Department of Materials Science, University of Salento, Lecce, Italy*

**Keywords:** Paracentrotus lividus, carbon nanoparticles, toxicity, 14-3-3 epsilon, gene expression

## Abstract

Members of the 14-3-3 protein family are involved in many important cellular events, including stress response, survival and apoptosis. Genes of the 14-3-3 family are conserved from plants to humans, and some members are responsive to UV radiation. Despite the high rate of pollution generated by nano-pollutants, up to now their toxic effect on development is totally obscure. Embryos treated with carbon nanoparticles, RNA preparation, retro-transcription and quantitative real-time PCR. In response to carbon nano-particles exposure, the embryos collected 24 h later showed a 3,07-fold at 5x10^12^ p and a 1,58-fold at 2.5x10^13^ p and a 1,92-fold at 2.5x10^14^ p increase in Pl14-3-3ε transcript levels compared with controls. The Pl14-3-3ε mRNA delocalization parallels the failure in archenteron elongation observed morphologically, as well as the lack of specific endoderm markers. Here, we report the isolation of the complete cDNA encoding the 14-3-3 epsilon isoform from Paracentrotus lividus sea urchin embryos, referred to as Pl14-3-3ε. Pl14-3-3ε mRNA levels were measured by RT-PCR during development and found to increase from the mesenchyme blastula to the prism stage. Our results confirm the involvement of 14-3-3ε in the stress response elicited by carbon nano-particles.

The term nano-pollution is generically referring to all waste generated during the fabrication of nanomaterials. These types of waste can be very dangerous because of their size, nanoparticles in the free state can be released into the air during production (or production accidents) or as waste, only to accumulate in the soil, in water or vegetable and thus be assimilated by the animals causing unknown effects. An environmental assessment of this phenomenon is very important because it could lead to define new environmental impacts. Currently one can not accurately predict or control the ecological impacts due to the releaseof these nano-pollutants in the environment. Ecotoxicological impacts of nanoparticles and the potential for bioaccumulation in plants and microorganisms are still under research.

In this regard, this work has examined the sea urchin Paracentrotus lividus, a model organism used in studies on embryonic development, and was exposed to the effects of carbon nanoparticles, synthesized according to the electrochemical method.

Members of the 14-3-3 protein family are involved in many important cellular events, including stress response, survival and apoptosis. Genes of the 14-3-3 family are conserved from plants to humans, and some members are responsive to UV radiation. Despite the high rate of their toxic effect on development pollution generated by nano-pollutants, up to now is totally obscure. Russo showed the isolation of the complete cDNA encoding the 14-3-3 epsilon isoform from Paracentrotus lividus sea urchin embryos, referred to as Pl14-3-3ε (1).

Proteins of the 14-3-3 family are considered as chaperones/adaptors, playing important roles in cellular homeostasis (2,3). Their involvement in stress response has been claimed as evidenced by the increased protein/mRNA levels following exposure to drugs and pesticides. For example, different subsets of 14-3-3 genes were induced after treatment with the fungal toxin fusicoccin in the tomato plant, demonstrating a correlation with the pathogen-associated defense response (4). In sponges, the 14-3-3 gene is induced by pesticides (PCB 118), in parallel with the increase in the levels of the heat shock protein 70 transcript, suggesting their role in preventing apoptosis (5). Under physiological conditions 14-3-3 homo- and hetero-dimers (6,7) can interact with a wide variety of signalling proteins, including the stress signalling BAD/BAX mitochondrial proteins and the FOXO transcription factor. This interaction is possible only if 14-3-3 proteins are un-phosphorylated and, by sequestering BAD, BAX and FOXO in the cytoplasm, their entrance into the mitochondrion/nucleus is prevented.

Following stress, 14-3-3 are phosphorylated by the JNK kinase and cannot bind the pro-apoptotic proteins, which are free to localize to their site of action, promoting apoptosis (8,9). Seven 14-3-3 isoforms (β, γ, ε, ζ, η, σ, τ) are known in mammals (8,10); up to 14 were described in the plant Arabidopsis thaliana (11), while two isoforms have been identified in yeast, Drosophila melanogaster and Bombyx mori (ε and ζ) (12,13). At least one isoform has been found in the sponge Geodia cynodium (5), and in the sea urchins Heliocidaris tuberculata, Heliocidaris erythrogramma (14). Three isoforms were annotated in the genome of Strongylocentrotus purpuratus, one referred to as ε, and two known as the isoforms 1 and 2 (15,16).

The sea urchin embryo is one of the most used marine invertebrates models for studying apoptosis, cellular stress and biochemical markers of pollution (17-22). It offers a suitable model for toxicological and developmental studies as the feeding larva (pluteus) is complete in about 48 h; the specification of the embryonic territories, including ectoderm, mesoderm and endoderm, begins as early as the 32- cells cleavage stage (23).

The aim of this study was to investigate the temporal and spatial expression of the 14-3-3ε gene in P. lividus sea urchin embryos, during development and in response to carbon nanoparticles treatment. The complete cDNA encoding Pl14-3-3ε was isolated by reverse transcriptase polymerase chain reaction (RT-PCR) from NPC exposed embryos mRNA. Quantitative real-time PCR (QPCR) analysis was used to assess the extent of gene expression.

## Material and methods

**Embryo culture and micromanipulation**

Adult Paracentrotus lividus sea urchins were collected along the coast of Salento. Eggs were fertilized and embryos cultured at a dilution of 4.000/mL in Millipore Filtered Sea Water (MFSW) containing antibiotics, at 16–18°C. Animals were induced to shed gametes by intracoelomic injection of 0.5 M KCl. Eggs were washed several times with filtered, natural seawater (SW), fertilized with a dilute suspension of sperm and cultured in SW in glass bowls.

**Morphological analysis**

The morphological analysis of perturbed and control embryos was performed using an inverted microscope Nikon Eclipse 80i, the images were recorded by a digital camera Nikon DMX 1200F.

**Treatments with carbon nanoparticles**

To a suspension of eggs fertilized were added in four different glass bowls i) control; ii)5×10^12^; iii) 2,5×10^13^; and iv)2,5×10^14^ carbon nanoparticles. Embryos at different developmental stages (6h, 24h) were collected by low-speed centrifugation.

**RNA preparation and relative RT-PCR analysis**

Total RNA was isolated according to the manufacturer’s instructions using TRIzol® Plus RNA Purification System (Invitrogen) from 25 collected embryos, frozen in liquid nitrogen and stored at -80°C until use. Briefly, total RNA (20μg) isolated from control and embryos treated with carbon nano-particles as described above were run on 1.5% agarose gel, under denaturing conditions (formamide 50%, MOPS 1X, formaldeide 5.5%). 

**Quantitative real-time PCR**

cDNAs were synthesized according to a single-step ThermoScript™ RT-PCR Systems kit (Invitrogen) protocol. About 0.1–1% of each RT reaction was used to run real-time PCR on a SmartCycler System (Cepheid) with SYBR^® ^Green JumpStart Taq ReadyMix (Sigma-Aldrich) and the primer pairs indicated in Table ​1. Real-time PCR samples were run in triplicate. Quantification measurements of gene expression were performed as described by the manual of Applied Biosystems Step One Plus real time PCR, a Comparative Threshold Cycle Method, using SYBR Green chemistry (24). Pl-S24 mRNA was used as the internal endogenous reference gene (25).

The QPCR was run as follow: 1X cycle denaturing 95°C for 10 min for DNA polymerase activation; 38 cycles: melting 95°C for 15 sec, annealing/extension 60°C for 60 sec.

**Synthesis of Carbon nanoparticles**

Carbon nanoparticles colloidal solution was obtained in the following. High purity graphite rod (SPI 99.99%) was used as an anode (5 mm diam), and a stainless steel rod (SWAGELOK AISI 1016, 5 mm diam) was used as a cathode. The electrodes were immersed in a becker containing deionized water (MILLIPORE MILLIP-Q 18.2 M) at a reciprocal distance of 10 mm. In the electrolysis process, the electric power applied to the electrodes was a constant voltage of 30 V. Simultaneously, the colloidal solution was forcedly dispersed by an ultrasonicator, which was the flat-type ultrasonic equipment (Flexonic-1200-35/72/ 100G, Mirae Ultrasonic Tech., Korea). To prevent the aggregation of nanoparticles, the ultrasonicator was continuously operated at the power output of 1000 W with the frequency of 100±5 kHz during the production of nanographite.

At the end of the reaction the solution of nanoparticles is diluted with distilled water (20ml solution of nanoparticles and 30 ml of water). The average size, size distribution, morphology and internal crystalline structure of the nanoparticles are studied through high resolution transmission electron microscopy, selected-area diffraction pattern and UV–Vis spectroscopic technique. 

**Physical analyses of carbon nanoparticles solution**

UV-visible spectra were recorded in the range between 200 and 800 nm using a T80 PG Instruments Ltd spectrophotometer. Optical spectra where obtained immediately after the synthesis and after 6-7 days by measuring the absorption of the prepared as in a quartz cuvette with a 1 cm optical path. 

Raman scattering measurements were obtained by back-scattering geometry with a RENISHAW spectrometer coupled to a LEICA metallographic microscope. An argon-ion laser operated at a wavelength of 514.5 nm and a 10mW incident power to avoid thermal effects provided excitation. Raman shifts were corrected by using silicon (111) reference spectra after each measurement. All spectra were recorded at room temperature.

Transmission Electron Microscopy (TEM) images and electron diffraction patterns were taken using an Hitachi 7100, at 100 kV representing the suitable acceleration voltage to obtain a good resolution and minimal radiation damage of the material. The specimens were prepared for transmission electron microscope observations by placing small droplets of colloidal solutions onto standard carbon supported 600-mesh copper grid and drying slowly in air naturally.

## Results

**UV–Vis absorption measurements**

**Fig 1:**
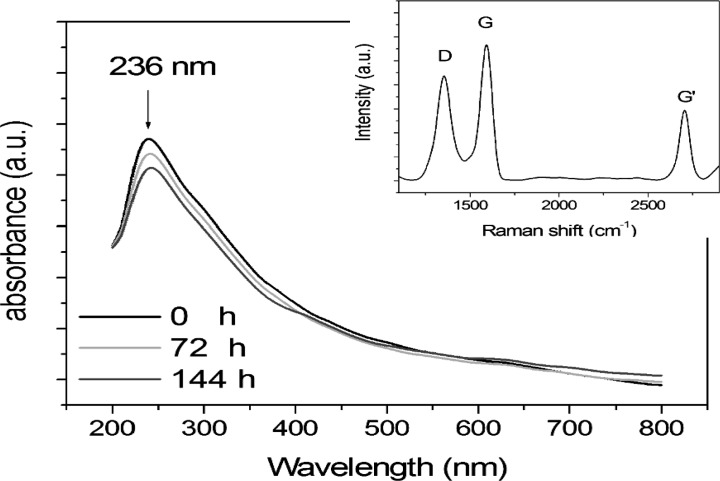
UV-vis absorption spectra of carbon nanoparticles immediately after the synthesis, after 72 and 144 hours. Related Raman spectrum (inset)

A typical UV–Vis spectra set recorded in the range between 200 and 800 nm immediately after the synthesis and after 72 and 144 hours is shown in Fig.1. The presence of a well defined peak at 236 nm is evident and also a shoulder at ~310 nm, that can be attributed to π→π* transitions of aromatic C-C bonds and n→π* transitions of C=O bonds, respectively, according to the experimental data reported for the unreduced nanocarbon dispersion (26,27).

It is interesting to note that the position of the peak remains unchanged over time, showing the stability of the nanoparticles dispersed in solution with a negligible aggregation and a sedimentation almost nothing.

In the inset of Fig.1 the Raman spectrum is reported. The peaks at about 1350, 1582, 1617 and 2700 cm^-1^ are assignable to carbon-based materials in low-dimensional configuration sp2 that is nanographite particles having dimensions of a few nanometers (28).

**Fig 2:**
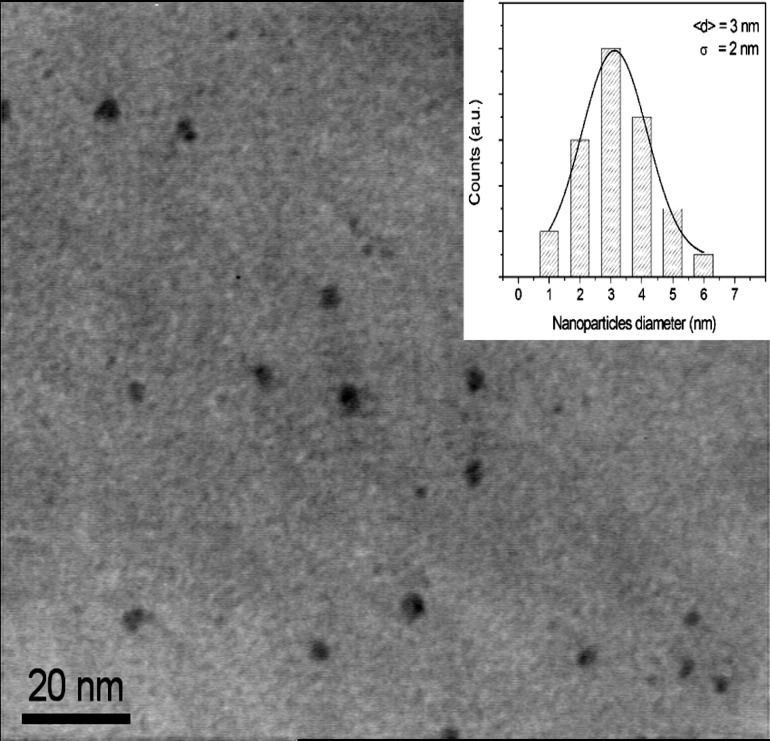
Transmission electron microscope image and histogram of silver nanoparticles size distribution (inset

To evaluate the exact size and structure of nanoparticles and to understand the aggregation process and the mechanism involved (29) a morphological analysis has been performed by Transmission Electron Microscopy (TEM). A typical TEM image is reported in Fig.2, it is evident that the carbon nanoparticles were almost spherical and were 3 nm great with a statistical standard deviation (ᵟ) of 2 nm as evident in the inset that report the histogram showing the size dispersion of observed carbon nanoparticles.

**Differential expression of Paracentrotus lividus 14-3-3ε mRNA in control embryos**

We performed quantitative PCR experiments with cDNA samples obtained by reverse transcription from total RNA extracted at different developmental stages of sea urchin embryos: unfertilized eggs as T0, T6 and T24. Quantitative measurements were performed using a comparative threshold cycle method, in which the Pl-S24 cDNA PCR product was used as an endogenous control gene, which was assumed to be constant during development.

**Fig 3:**
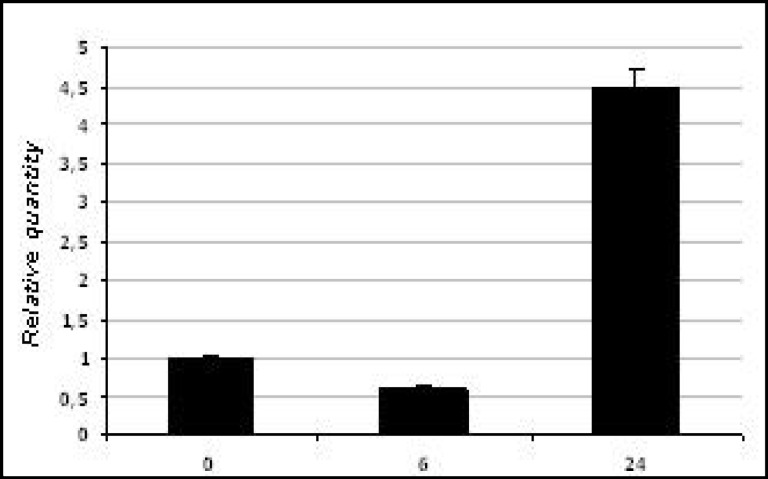
Quantitative PCR analysis of the P. lividus 14-3-3ε transcription levels in sea urchin embryos at different developmental stages, showed in the lower panel: eggs, morula, late gastrula, 0, 6, 24 hours post-fertilization. Pl-S24 was used as normalizing endogenous gene; cDNA extracted from eggs was used as reference sample and assumed as 1 in the histogram. Each bar represents the mean of three independent experiments ±SD. Mean values were significantly different according to the one-way ANOVA (P<0.05).

The egg cDNA was used as reference sample and was assumed as 1 in arbitrary units. The histogram in Fig.3 shows the Pl14-3-3ε transcript levels during sea urchin development: the relative quantity fold ranged from 0,6 at 6h after fertilization to 4.5 at the late gastrula phase (24h after fertilization).

We selected the morula stage and late gastrula stage for measure Pl14-3-3ε transcript levels after carbon nanoparticles exposure. Previous study, infact, demonstrated lower levels between eight cell stage and morula, as well as at the pluteus stage. Therefore, it seems that during the period between fertilization and morula, no mRNA synthesis is required, as the transcripts detected in the unfertilized egg are probably used during the first developmental stages. Moreover, the 32 cell stage is a crucial period during development and represents an ideal stage to study stress effects. The histogram summarizes results of three independent QPCR analyses.

**Morphology in control and carbon nanoparticles exposed embryos**

The sea urchin Paracentrotus lividus is a classical model for ecotoxicological studies due to the particular sensitivity to environmental events 

related to fertilization and development. In this experiment the toxicity of a solution of carbon nanoparticles was evaluated by testing three different doses 5×10^12^ p, 2.5×10^13^ p and 2.5×10^14^ p with a period of light/dark and controlled flow of oxygen and under aseptic conditions, while a control was carried out at the same time. The events that characterize the stages of fertilization until the early stagesof development were checked at the inverted optical microscope.

The fertilized egg undergoes a first division meridian gives rise to two equal blastomeres. In the control observed that 90% of fertilized eggs follows a normal division, while the remaining 10% produces asymmetrical blastomeres. 

**Fig 4:**
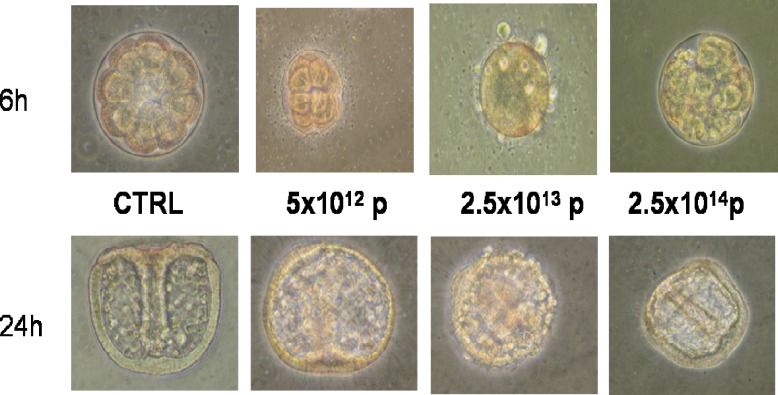
Embryos incubated at 6 and 18 hours in the presence of carbon nanoparticles

In NPC treated with low amounts of 70% of the cells are dividing, two blastomeres are observed and remain equal to the membrane around the sperm fertilization. the percentage of dividing cells with normal increases in the treatment medium and high concentration, 77% and 82.25%, respectively. At the same time is observed for each treatment asymmetrical percentage of dividing cells, in particular the 30% treated with low amounts of NPC, 20.5% in the treated medium in quantity and 17.59% treated with high amounts. After 4-6hours in the samples treated with different concentrations of NPC, 5x10^12^ p, 2.5x10^13^ p and 2.5x10^14^ p observed respectively 65%, 60% and 70% of normal morula. Up to 48 hours, the relationship between normal embryos and modified remain unchanged under all conditions set up. The normal embryos proceed in development to the formation of gastrulae, while the altered embryos progressing to the stage of blastula, however, characterized by altered structures (Fig.4). 

**Expression of Pl14-3-3** **ε** **in control and carbon nanoparticles exposed embryos**

**Fig 5:**
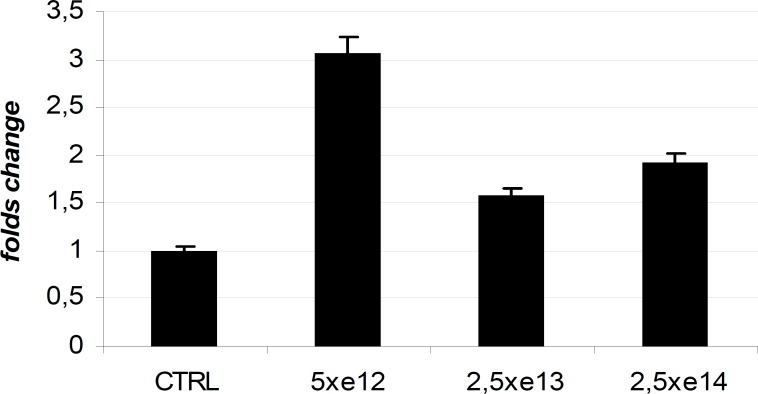
RT-PCR of the Pl-14-3-3 epsilon gene in embryos incubated at 24 hours without and with the presence of carbon nanoparticles

Quantitative analysis of Pl14-3-3ε specific transcript, normalized to Pl24S rRNA, was performed by reverse transcriptase (RT) real-time PCR. In order to investigate that the carbon nanoparticles had an effect on the Pl14-3-3ε gene, mRNA expression levels were investigated by QPCR in control and carbon nanoparticles exposed embryos at the doses of 5x10^12^ p, 2.5x10^13^ p and 2.5x10^14^ p. The CTRL cDNA was assumed as 1 in arbitrary units. The histogram in Fig.5 shows the Pl14-3-3ε transcript levels at the late gastrula phase (24h after fertilization). It is possible to observe an increment of 3,07 folds in the embryos incubated with 5x10^12^p, of 1,58 folds in the embryos incubated with 2,5x10^13^p, of 1,92 folds in the embryos incubated with 2,5x10^14^p. RT-PCR results are in agreement with the statistics obtained by morphological observation of the embryos. This is observed in the various treaties that embryos grown in the presence of low concentrations of carbon nanoparticles are most damaged, and the gene Pl-14-3-3-epsilon is more expressed, because of its involvement in response to stress factors. Instead of embryos grown with medium and high concentrations of nanoparticles show damage less obvious and less increase of Pl-14-3-3 epsilon gene.

## Discussion

The Paracentrotus lividus is one of excellence in animal models to study water pollution and, in general, the environment. the rate of toxicity induced by nanoparticles allows to analyze changes in cell behavior from the earliest stages of development until the formation of larva. During this period the orgasnism is particularly delicate because they occur important biochemical and physiological changes. any contact with toxic substances, could result in slowing down or speeding up the development or the development is not correct and/or death of the organism, or a subsequent accumulation of toxic substances during larvale. Our results suggest that the carbon nanoparticles cause changes (asymmetric divisions) and acceleration of development between two and four hours. These irregular behavior, although clearly visible in all three treatments are much more evident in the treatment 5x10^12^p. These results are in agreement with literature data: In fact, Nel (30) has demonstrated the toxicity of carbon nanotubes in mammalian cells suggesting oxidative stress as the most likely mechanism to explain the toxicity. This study has highlighted the negative effects of carbon nanoparticles in aquatic species and, therefore, we believe that we should pay particular attention to all applications involving the use of carbon nanomaterials.

In conclusion, we have demonstrated a direct relationship between carbon nanoparticles exposure of sea urchin embryos and Pl14-3-3ε mRNA levels, suggesting its implication in the regulative cascade activated in the stress response. To the best of our knowledge this is the first time that a carbon nanoparticles induced 14-3-3ε transcriptional regulative mechanism has been demonstrated. As previously described, upon stress 14-3-3 proteins are phosphorylated by the JNK kinase and are thus unable to bind BAD, BAX and FOXO, which are then able to translocate to mitochondria and nuclei, exerting their pro-apoptotic functions (31,32). The increase in Pl14-3-3ε mRNA detected after NPC stress could result in higher 14-3-3 protein levels (possibly not phosphorylated) which could then bind a large quantity of the above mentioned factors, determining cellular survival.

On the basis of studies described by Russo et al. 2010 we confirmed the use of sea urchin embryos for examining the role of 14-3-3 in cell stress response pathways. Finally, 14-3-3ε could be used as a valuable molecular biomarker to identify the dangerous effects of sunlight occurring in marine organisms living in shallow waters.

In this study, we observed the transcriptional response of gene 14-3-3 in Paracentrotus lividus, and provide data to demonstrate its response to carbon nanoparticles showing that a clear correlation exists between nanoparticles exposure and the transcriptional regulation of this gene. Previous studies showed a rapid induction of 14-3-3’s in tomato plant roots deprived of iron (33). This is particularly interesting due to the close coupling of copper and iron metabolism (34), demonstrated by their shared cell surface reductases Fre1 and Fre2. Direct evidence for the role of 14-3-3 in stress response has been shown in the macro-alga Fucus vesiculosus (35). 
